# Size matters: An observational study investigating estimated height as a reference size for calculating tidal volumes if low tidal volume ventilation is required

**DOI:** 10.1371/journal.pone.0199917

**Published:** 2018-06-29

**Authors:** Benjamin Sasko, Ulrich Thiem, Martin Christ, Hans-Joachim Trappe, Oliver Ritter, Nikolaos Pagonas

**Affiliations:** 1 Department of Cardiology, Brandenburg Medical School, University Hospital Brandenburg, Germany; 2 Department of Cardiology, Marienhospital Herne, Ruhr-University Bochum, Herne, North Rhine-Westphalia, Germany; 3 Department of Medical Informatics, Biometry and Epidemiology, Ruhr-University Bochum, Bochum, North Rhine-Westphalia, Germany; 4 Department of Nephrology, Marienhospital Herne, Ruhr-University Bochum, Herne, North Rhine-Westphalia, Germany; National Yang-Ming University, TAIWAN

## Abstract

**Purpose:**

Acute lung injury is a life threatening condition often requiring mechanical ventilation. Lung-protective ventilation with tidal volumes of 6 mL/kg predicted body weight (PBW, calculated on the basis of a patient’s sex and height), is part of current recommended ventilation strategy. Hence, an exact height is necessary to provide optimal mechanical ventilation. However, it is a common practice to visually estimate the body height of mechanically ventilated patients and use these estimates as a reference size for ventilator settings. We aimed to determine if the common practice of estimating visual height to define tidal volume reduces the possibility of receiving lung-protective ventilation.

**Methods:**

In this prospective observational study, 28 mechanically ventilated patients had their heights visually estimated by 20 nurses and 20 physicians. All medical professionals calculated the PBW and a corresponding tidal volume with 6 ml/kg/PBW on the basis of their visual estimation. The patients’ true heights were measured and the true PBW with a corresponding tidal volume was calculated. Finally, estimates and measurements were compared.

**Results:**

1033 estimations were undertaken by 153 medical professionals. The majority of the estimates were imprecise and resulting data comprised taller body heights, higher PBW and higher tidal volumes (all p≤0.01). When estimates of patients´ heights are used as a reference for tidal-volume definition, patients are exposed to mean tidal volumes of 6.5 ± 0.4 ml/kg/PBW. 526 estimation-based tidal volumes (51.1%) did not provide lung-protective ventilation. Shorter subjects (<175cm) were a specific risk group with an increased risk of not receiving lung protective ventilation (OR 6.6; 95%CI 1.2–35.4; p = 0.02), while taller subjects had a smaller risk of being exposed to inadequately high tidal volumes (OR 0.15; 95%CI 0.02–0.8; p = 0.02). Furthermore, we found an increased risk of overestimating if the assessor was a female (OR 1.74; 95%CI 1.14–2.65; p = 0.01).

**Conclusion:**

The common practice of visually estimating body height and using these estimates for ventilator settings is imprecise and potentially harmful because it reduces the chance of receiving lung-protective ventilation. Avoiding this practice increases the patient safety. Instead, height should be measured as a standard procedure.

## Introduction

Acute lung injury (ALI) is a life-threatening condition with severe hypoxaemia that typically necessitates treatment in the intensive care unit (ICU) and mechanical ventilation. Lung-protective ventilation with low tidal volumes (6 ml/kg predicted body weight [PBW]) and plateau pressures of ≤30 cmH_2_O have been shown to decrease mortality and are, therefore, part of a ventilation strategy [1]. A prospective cohort study published in 2012 evaluated the association of ventilator tidal volume with two-year survival in patients with ALI [2]. In this study, a three-level categorical model was used to compare three different mean tidal volumes, <6.5, 6.5–8.5, and >8.5 ml/kg PBW, and their impact upon survival. Compared with a mean tidal volume <6.5 ml/kg/PBW, the adjusted hazard ratio for two-year mortality were 1.59 for a mean tidal volume of 6.5–8.5 ml/kg/PBW and 1.97 for >8.5 ml/kg/PBW. The same study also demonstrated an 18% relative increase in mortality for each 1-ml/kg/PBW increase in mean tidal volume [2]. Clearly, a precise definition of tidal volumes is crucial to achieve a respiratory support with low tidal ventilation (LTV) of <6.5ml/kg/PBW. The current recommended ARDSnet formula uses body height as the only changeable variable in a mathematical equation to calculate the PBW, upon which tidal volumes are finally defined [1]. Therefore, the prerequisite for calculation of the PBW is the knowledge of an exact body height. Hence, an accurate measurement of height is necessary to provide an optimal ventilation strategy in ALI.

Several studies have focused on the actual effect of a LTV strategy itself or on how stringently LTV is applied [3,4], but the question of height determination itself has not been studied comprehensively. Unfortunately, most studies investigating LTV strategies do not describe what reference heights were used to calculate and define the target tidal volumes [5]. The technique to obtain a reference height, however, is crucial because the definition of tidal volumes is reliant on accurate information concerning body height. There are few data on the quality of measurement of body height, but as indicated by some evidence, it is common practice for intensivists to visually estimate body height [6–8]. A UK survey investigating the calculation of tidal volumes revealed that only one-third of all tidal volumes are defined as recommended by guidelines. In fact, it is likely that most ICUs use visual estimates to calculate tidal volumes, a method which is apparently becoming common practice in intensive care therapy [7]. Other studies have suggested measurement of forearms, lower legs or demispan to calculate tidal volumes [9,10].

Height estimations are known to be potentially imprecise, but ICU clinicians seem to use estimates as a reference figure when accurate information is needed [5,7]. Visual estimation of body height can be done at a glance during busy ward rounds or in primary care and is a tempting option due to time constraints [8]. We investigated the accuracy of height estimates as a reference for the definition of tidal volumes and compared the results with the real data of body measurement and lung-protective tidal volumes. We hypothesised that, by visual estimation of body height, patients might be exposed to tidal volumes exceeding the recommended thresholds of lung-protective ventilation.

## Material and methods

This non-interventional, observational study aimed to investigate the practice of visual estimation of body height, followed by a simulation of ventilator settings with low tidal volumes. Between February and April 2016, 28 patients receiving mechanical ventilation treated in the ICU of a tertiary centre (Marienhospital Herne, University of Bochum, Germany) were enrolled prospectively into this study. Inclusion criteria were age ≥18 years and mechanical ventilation on a pressure-controlled mode in the supine position. Exclusion criteria were body height <150 cm or > 200 cm, amputation of lower limbs and colonisation with multi-resistant pathogens. The study was registered and approved by the institutional review board (Ethics Committee of Ruhr-Universität Bochum, 15-5414-BR) and performed in accordance with the Declaration of Helsinki and its later amendments. It was retrospectively registered in the German Clinical Trial Register (DRKS), trial number DRKS00010899. Informed consent was obtained from all patients post hoc after extubation or from relatives.

### Visual estimation and measurement of body height

We asked medical professionals working in the ICU of our hospital (nurses, physicians, respiratory therapists) to visually estimate the body sizes of mechanically ventilated patients. Time of estimation was the first and third Friday of each month during the 3-month observational period. All mechanically ventilated patients at the time of the morning ward round were included in our study if they were eligible. Each ventilated patient was scheduled to be estimated by 40 assessors (20 physicians and 20 nurses or respiratory therapists).

Visual estimation of height was done in the supine position. All ventilated patients were sedated with sufentanil and midazolam or propofol (Richmond Agitation–Sedation Scale, −3/−4). Assessors were unaware of the patients’ true body heights or weights. Estimations were done independently without the help of a second person. No tools were allowed for measurement. Data were collected anonymously using predefined reporting forms. Assessors were asked to provide information about their sex, age, profession, and years of experience.

The authors undertook an exact crown-to-heel measurement of each patient’s height using a 255-cm measuring tape. After each measurement, the tape was disinfected (Kohrsolin® FF 1.0% tissues).

### Ascertainment of predicted body weight and simulation of ventilator settings

Based on their visual estimations of body height, assessors were asked to define a PBW and a corresponding tidal volume with 6 ml/kg/PBW, using their estimated height as a reference. By that, we simulated a lung protective ventilator setting and generated estimates of PBW.

To compare the results of estimates with true measures, the authors calculated the precise PBW using the measured height and standard formula from the ARDSNet study [1]:

PredictedBW(males)=50+0.91(cmofheight‑152.4)

PredictedBW(females)=45.5+0.91(cmofheight‑152.4)

To demonstrate the impact of magnitude estimation on the quality of mechanical ventilation, we performed a simulation of ventilator settings: using the estimated and calculated PBW, a corresponding tidal volume with 6 ml/kg/PBW was calculated by the authors. As a result, two data sets were generated: estimated magnitudes (height, PBW, tidal volumes) and measured magnitudes (height, PBW, tidal volumes).

As a final step, the authors divided each estimated tidal volume by the true PBW. In this way, we generated the equivalent and “real” tidal volume that the patient would be exposed to if visual estimation of body height was undertaken and used as a reference height for tidal volumes (hence described as “exposed tidal volumes”):

Exposedtidalvolume(ml/kgPBW)=estimatedtidalvolume/(calculated)PBW

We classified the exposed tidal volumes in a three-level categorical model (<6.5, 6.5–8.5, and >8.5 ml/kg/PBW) introduced previously by Needham and co-workers [2].

**Fig 1:**
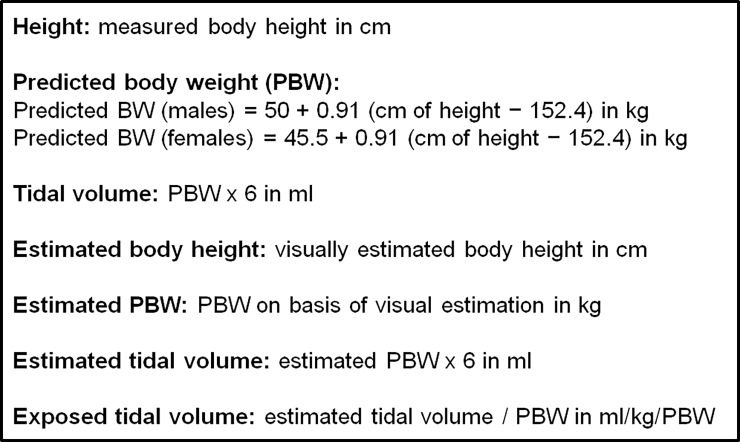
Definition of variables: Summary of all calculated and estimated variables.

All estimations and calculations were performed as a simulation and therefore only serve as a theoretical ventilation model. All variables used in this study are summarized in Fig 1.

### Secondary analysis

Assessors were asked to provide estimates of body height and estimates of PBW without any technical specifications given prior by the authors. It therefore remains unclear how frequently the correct ARDSnet formula was used by the assessors to calculate the PBW or if rounded figures were used. To illustrate the effect of estimated height but correctly calculated PBW, we performed a secondary analysis and repeated all calculations of PBW by using the correct ARDSnet formula to provide a second set of tidal volumes [1]:

PredictedBW(males)=50+0.91(cmofheight‑152.4)

PredictedBW(females)=45.5+0.91(cmofheight‑152.4)

In this way, we generated a tidal volume that the patient would be exposed to if visual estimation of body height was undertaken, but the correct ARDSnet formula was used to calculate tidal volumes. As described above, a corresponding tidal volume with 6 ml/kg/PBW was calculated in the following and we again divided each estimated tidal volume by the true PBW. Here too, the resulting exposed tidal volumes were classified in a three-level categorical model (<6.5, 6.5–8.5, and >8.5 m/kg/PBW).

This additional analysis illustrates the effect if the only estimation performed would be the estimation of height.

### Statistical analyses

Categorical variables are summarised as proportions. Continuous data are described by mean or median values if they have a non-normal distribution, as well as by range and standard deviation (SD). For comparison of continuous data the Mann–Whitney or Wilcoxon rank-sum test were used, as appropriate. To study the relationship between true body height and distribution of estimated tidal volumes ≥6.5 ml/kg/PBW, the true body height was divided into several strata at 5-cm intervals.

A shorter stature is known to be a risk factor for underuse of lung-protective ventilation strategies [11], so we undertook further analyses in two subgroups: true body height was used as a variable for dichotomous classification. After carrying out descriptive analysis, a threshold of <175 cm for the first subgroup was chosen post hoc according to the median of our study population, and referred to the height distribution within our study group.

To quantify the agreement between the measurement methods of visual estimation with the ‘gold standard’ of height measurement, a modified Bland–Altman plot was created. In this graphical analysis, we plotted the differences between both methods against the true height. In contrast to a ‘classic’ Bland–Altman plot, our modified plot used the true height as a variable for the x-axis and not the mean values of a measurement method.

A logistic regression model was build to identify assessors’ characteristics that could have an impact on the results of height estimation, with adjustment for sex, age, profession and years of work experience.

For all analyses, P<0.05 was considered significant. Analyses were carried out using Stata\IC v13.1 (Stata, College Station, TX, USA).

## Results

In the 3 months of data collection, 28 patients were enrolled prospectively. During this period, 1033 estimations were made by 153 assessors, resulting in mean of 6.7 estimations per assessor.

### Missing data

Each patient was supposed to be evaluated by 40 assessors, so a maximum of 1120 estimations could be obtained. For four patients (14.3%) developing multi-resistant pathogens during the study visual assessments could not be completed, while in another four patients (14.3%) the planned number of 40 assessors could not be achieved due to organizational obstacles during daily patient care. Thus, 87 estimations were not obtained.

### Baseline data

Baseline characteristics of patients are presented in Table 1. Men were significantly taller than women by a mean of ≈12 cm (174.9 *vs*. 164.2 cm, p = 0.015). Twenty-two patients were male (78.6%).

**Table 1:** Baseline characteristics of patients.

	Total (n = 28)	Male (n = 22)	Female (n = 6)
Age (years)	64.8 ± 8.9	65.5 ± 9.3	62.3 ± 7.4
Body height (cm)	172.6 ± 9.4	174.9 ± 8.6	164.2 ± 7.6
Estimated body height (cm)	177.0 ± 7.6	179.1 ± 6.0	169.5 ± 8.1
Predicted body weight (kg)	67.5 ± 9.4	70.5 ± 7.8	56.9 ± 6.7
Estimated predicted body weight (kg)	73.9 ± 7.5	76.1 ± 5.5	65.9 ± 9.1

### Main results

Baseline body height was measured crown-to-heel by the authors, and the corresponding PBW was calculated for all 28 patients. In addition, a lung-protective tidal volume with 6 ml/kg/PBW was calculated by the authors (Table 2). The study group had a mean body height of 172.6 ± 9.4 cm, mean PBW of 67.9 ± 9.3 kg, and mean theoretical tidal volume of 407 ± 57.0 ml (6.0 ml/kg of PBW = 6.0 x 67.9).

**Table 2:** Distribution of body height, predicted body weight, tidal volume, and estimated tidal volume.

	Total (n = 28)	<175 cm (n = 14)	≥175 cm (n = 14)	p
Body height (cm)	172.6 ± 9.4	165.2 ± 6.8	180.0 ±4.3	0.004
Estimated body height (cm)	177.0 ± 7.5	171.7 ± 6.8	182.4 ±3.2	
Mean difference (cm)	4.8 ± 4.7	7.2 ± 4.8	2.3 ± 3.1	
Mean difference (%)	2.7	4.4	1.2	
Predicted body weight (kg)	67.9 ± 9.3	60.6 ± 7.3	75.2 ± 3.9	0.002
Estimated (kg)	73.9 ± 7.5	68.9 ± 7.7	78.9 ± 2.6	
Mean difference (kg)	6.0 ± 4.1	8.3 ± 4.1	3.7 ± 2.7	
Mean difference (%)	8.8	13.6	4.9	
Tidal volume (ml)	407 ± 57.0	362 ± 43.3	452 ± 24.5	0.002
Estimated tidal volume (ml)	441 ± 46.7	409 ± 45.9	473 ± 16.0	
Mean difference (ml)	34 ± 23.6	47 ± 23.9	21 ± 15.3	
Mean difference (%)	8.3	12.9	4.6	
Exposed tidal volume (ml/kg/PBW)	6.5 ± 0.4	6.8 ± 0.4	6.3 ± 0.2	0.001

Measured *vs*. estimated values are presented for all included subjects (n = 28) and after dichotomous classification (threshold <175 cm). For easier interpretation, differences between visual estimates and measured magnitudes are expressed as absolute values (cm, kg, ml) and percentage of measured values. p-values refer to the mean difference of absolute values.

Table 2 compares the measured baseline characteristics with the results of visual estimations of body height, PBW and tidal volumes for both, the total study population and the subgroups (<175 cm and ≥ 175cm). In summary, the mean of all estimates resulted in a higher body height (mean difference, 4.8 ± 4.7 cm), higher PBW (6.0 ± 4.1 kg) and higher tidal volume (34 ± 23.6 ml).

Estimates for patients of body height <175 cm resulted in a higher mean estimated tidal volume (6.8 ± 0.4 ml/kg/PBW), while in contrast estimates for patients with a body height ≥175 cm resulted in tidal volumes of <6.5ml/kg/PBW (6.3 ± 0.2 ml/kg/PBW), p = 0.001. As a result, shorter subjects were a specific risk group with an increased risk of not receiving LTV (OR 6.6; 95%CI 1.2–35.4; p = 0.02), while taller subjects benefited from their underestimation of height, resulting in a smaller risk of being exposed to inadequately high tidal volumes (OR 0.15; 95%CI 0.02–0.8; p = 0.02).

**Fig 2:**
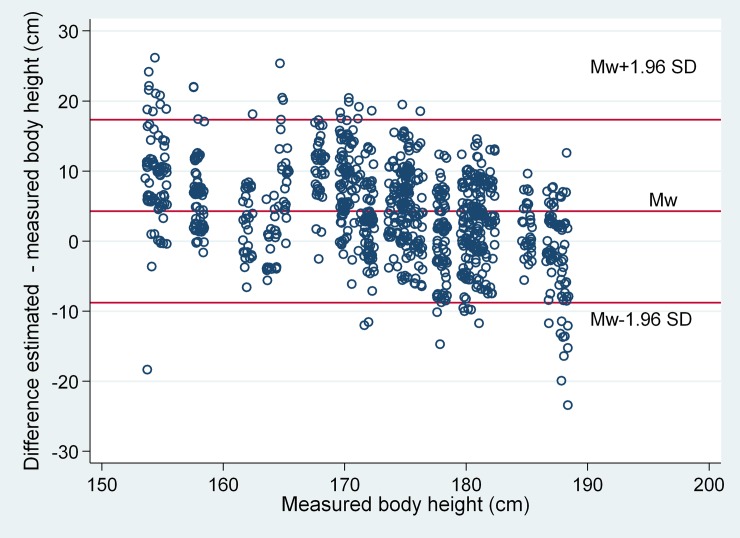
Modified Bland–Altman plot: Each dot represents an estimate of body height. Differences between measured and estimated height (y-axis) are plotted against the true height (x-axis).

The modified Bland–Altman plot (Fig 2) illustrated the discrepancies between measured and estimated body heights with a clear height-dependent trend: patients with a body height of <175 cm were frequently overestimated and rarely underestimated.

### Categorisation of estimated tidal volumes

Estimated tidal volumes were classified into three categories: <6.5, 6.5–8.5, and >8.5 ml/kg/PBW. After classification, 503 out of 1033 ventilator settings (48.9%) were assigned to the category <6.5 ml, whereas 51.1% of 526 ventilator settings were allocated to 6.5–8.5 ml/kg or >8.5 ml/kg groups (Table 3).

**Table 3:** Distribution of estimates and categorisation of exposed tidal volume within body-height strata.

Measured body height (cm)	Estimated body height	Predicted body weight	Estimated predicted body weight	Exposed tidal volume (ml/kg/PBW)		
				<6.5	6.5–8.5	>8.5
<155 (n = 40)	164.2 ± 8.0	51.5 ± 0	63.0 ± 0	8 (20.0%)	29 (72.5%)	3 (7.5%)
155–159 (n = 112)	164.1 ± 5.1	50.6 ± 3.6	61.1 ± 1.2	20 (18.0%)	86 (77.5%)	5 (4.5%)
160–164 (n = 56)	164.4 ± 4.6	57.4 ± 1.9	60.0 ± 0.4	40 (72.7%)	15 (27.3%)	0
165–169 (n = 64)	177.2 ± 5.2	62.6 ± 2.2	74.2 ± 0.1	7 (10.9%)	55 (85.9%)	2 (3.1%)
170–174 (n = 223)	177.3 ± 5.7	67.4 ± 1.3	75.9 ± 3.5	88 (39.5%)	135 (60.5%)	0
175–179 (n = 228)	179.8 ± 5.7	71.6 ± 1.3	76.8 ± 1.0	120 (52.6%)	108 (47.4%)	0
180–184 (n = 196)	183.1 ± 5.8	75.8 ± 0.8	79.6 ± 2.7	126 (64.6%)	69 (35.4%)	0
≥185 (n = 114)	185.9 ± 5.9	81.1 ± 1.4	81.5 ± 0.9	94 (83.2%)	19 (16.8%)	0
Total (n = 1033)				503 (48.9%)	530 (51.1%)	

In accordance with our findings regarding the distribution of body height, PBW and tidal volumes, the patient stature played a major role. Table 3 suggests that tidal volumes >8.5 ml/kg PBW were calculated only for patients of height <170 cm. Furthermore, the vast majority of all calculated tidal volumes 6.5–8.5 ml/kg/PBW occurred in patients of height <175 cm.

### Results from secondary analysis

As seen in Table 4, the estimation of height and the calculation of PBW using the ARDSnet formula results in lower PBW (71.9 ± 9.1 vs. 73.9 ± 7.5) and lower tidal volumes (431 ± 54.6 vs. 441 ± 46.7) if compared with results from estimation of both, height and PBW (Table 2), p all <0.001. Furthermore, the results suggest that calculating the PBW with the use of estimated height and the correct ARDSnet formula reduces the extent of non-adherence to lung-protective ventilation by approximately 25% (51.1% vs. 39.1%, Table 3 and Table 5).

**Table 4:** The use of measured height and the ARDSnet formula to calculate predicted body weight and tidal volumes.

	Total (n = 28)	p
Body height (cm)	172.6 ± 9.4	
Estimated body height (cm)	177.0 ± 7.5	
Predicted body weight (kg)	67.9 ± 9.3	
Estimated (kg)	71.9 ± 9.1	
Mean difference (kg)	4.0 ± 4.1	<0.001
Mean difference (%)	5.6	
Tidal volume (ml)	407 ± 57.0	
Estimated tidal volume (ml)	431 ± 54.6	
Mean difference (ml)	24 ± 36.5	<0.001
Mean difference (%)	5.6	
Exposed tidal volume (ml/kg/PBW)	6.3 ± 0.6	<0.001

Results of using estimated height and the ARDSnet formula to calculate PBW and tidal volumes in all cases. For easier interpretation, differences between visual estimates and measured magnitudes are expressed as absolute values (cm, kg, ml) and percentage of measured values. p-values refer to the mean difference of absolute values.

**Table 5:** Combination of estimated height and ARDSnet formula to calculate PBW and tidal volumes.

Measured body height (cm)	Estimated body height	Predicted body weight	Estimated predicted body weight	Estimated tidal volume (ml/kg/PBW)		
				<6.5	6.5–8.5	>8.5
<155 (n = 40)	164.2 ± 8.0	51.5 ± 0	61.5 ± 5.8	2 (5.0%)	36 (90.0%)	2 (5.0%)
155–159 (n = 112)	164.1 ± 5.1	50.6 ± 3.6	57.7 ± 6.3	35 (31.3%)	77 (68.7%)	0
160–164 (n = 56)	164.4 ± 4.6	57.4 ± 1.9	56.3 ± 4.1	53 (94.6%)	3 (5.4%)	0
165–169 (n = 64)	177.2 ± 5.2	62.6 ± 2.2	72.6 ± 4.7	5 (7.8%)	59 (92.2%)	0
170–174 (n = 223)	177.3 ± 5.7	67.4 ± 1.3	72.2 ± 5.2	134 (60.1%)	89 (39.9%)	0
175–179 (n = 228)	179.8 ± 5.7	71.6 ± 1.3	74.5 ± 5.2	151 (66.2%)	77 (33.8%)	0
180–184 (n = 196)	183.1 ± 5.8	75.8 ± 0.8	77.9 ± 5.3	145 (74.0%)	51 (26.0%)	0
≥185 (n = 114)	185.9 ± 5.9	81.1 ± 1.4	80.5 ± 5.4	104 (91.2%)	10 (8.8%)	0
Total (n = 1033)				629 (60.9%)	404 (39.1%)	

### Characteristics of assessors

A total of 153 assessors participated in our study, of which 108 persons (70.6%) completed a questionnaire. Missing data were found for the variables ‘sex’ in 12 cases (7.8%), ‘age’ in 22 cases (14.4%), ‘profession’ in 14 cases (9.2%) and ‘years of experience’ in 41 cases (26.8%).

Seventy-five (53.1%) assessors were female. Mean age of all assessors was 34.5 ± 7.8 years. Seventy-five assessors (56.8%) were physicians, with 50 (66.6%) being residents. A mean of 13.4 ± 8.1 years of work experience was found within the group, showing a distribution of: 28 assessors (25%) with <8 years, 26 assessors (23.2%) with ≥8 years, 27 assessors (24.1%) with ≥12 years and 31 assessors (27.6%) with ≥16 years of work experience.

A logistic regression model adjusted for sex, age, profession and years of work experience revealed a 1.74-fold increased risk of overestimating if the assessor was a female (OR 1.74; 95%CI 1.14–2.65; p = 0.01). Other variables (age, profession, years of work experience) did not affect the results of estimating the body height.

## Discussion

In our observational study which evaluated 1033 visual estimates of body height of 28 mechanically ventilated patients we identified such practice of body-size definition as being imprecise and potentially harmful. Most estimates were significantly inaccurate and deviated from measured results.

This study offers three principal findings: firstly, as visualized in the modified Bland-Altman plot, it became obvious that the accuracy of the estimates was dependent on the actual body height: small heights were frequently overestimated, while tall heights were frequently underestimated. Overall, we found poor agreement between estimated heights and actual body height, especially in patients with either a short or tall stature. Secondly, we offer an explanation for this poor agreement: estimates were biased towards the mean value of the underlying sample distribution, a statistical phenomenon known as ‘regression effects’ [12]. Thirdly, this systematic bias in magnitude estimation (body height, PBW, tidal volume) is a potential hazard when low-tidal ventilation is required, especially in patients with a shorter stature (<175cm).

The potential risk of inaccurate visual height estimations can be demonstrated by translating estimated height into a PBW and a corresponding lung tidal volume: what assessors expected to be a lung protective low-tidal volume with 6 ml/kg/PBW, actually resulted in an equivalent (mean exposed) tidal volume of 6.5 ± 0.4 ml/kg/PBW. Estimates therefore exceeded the target tidal volume by an average of 0.5 ml/kg/PBW (+ 8.3%). As summarized in Table 2, our results indicate that the extent of body-size misjudgement was dependent upon the patient´s true body height: shorter patients, in this study <175cm, were more frequently overestimated and had a 6.6-fold increased risk for an estimated tidal volume of ≥6.5 ml/kg/PBW. As a conclusion, shorter patients (<175cm) are a specific risk group for not receiving LPV. This is an interesting observation because one would rather assume that a larger body mass (obesity, tall height) would generally also lead to overestimation of height and result patient´s increased risk of inadequately high tidal volumes.

In the light of the results published by Needham and co-workers [2], our data suggest that under “real-life” conditions half of our estimates would lead to an increased risk of mortality, as 51% of all estimates exceeded the threshold of 6.5 ml/kg/PBW. The results of our secondary analysis show that the use of the ARDSnet formula does improve the results and reduces the proportion of inadequate tidal volumes by approximately 25% (Table 5). In spite of using the ARDSnet formula, it remains a high proportion of non-adherence to lung-protective tidal volumes: as Table 5 indicates, 39.1% of all estimates resulted in a tidal volume of at least 6.5 ml/kg/PBW. These findings illustrate that estimating a single parameter (height) is better than estimating two parameters (height and PBW). None the less, this method is still associated with suboptimal care because of the potential risk of inadequate ventilation.

As a conclusion, our findings confirm the hypothesis that visual estimation of height is inaccurate and potentially harmful if used as a reference height for ventilation settings. The technique of height estimations may reduce the chance of receiving lung-protective ventilation. Our study addresses the obvious discrepancy between recommended practice and clinical reality. The estimation of height seems to be a popular shortcut in daily routine, but its impact on the quality of ventilation has not been studied so far–especially when accurate tidal volumes are needed, e.g. for ARDS patients. Ironically, the simple measure of height is easily applicable in clinical routine without any budget impact. At the same time, it reduces the risk for patients of not receiving lung-protective ventilation. There is no use discussing potential benefits of elaborate ventilation treatment strategies if the very basic requirements for a successful ventilation therapy are frequently not observed.

This is the first study to investigate the accuracy of the definition of ‘visual tidal volume’ and its impact upon the quality of mechanical ventilation. Our findings offer a new perspective with regard to high mortality in ALI that has not been discussed so far. That is, intensivists may be unaware of the fact that patients do not receive lung-protective ventilation and may not realise a basic error in magnitude estimation with respect to definition of tidal volume. It is conceivable that ventilator settings are labelled ‘lung protective’ and the results regarding outcome are, therefore, also biased. We believe that these results may help to sensitize physicians with regard to this issue and result in a less frequent use of estimations in clinical daily routine. In this way our study could contribute to the increase of patient safety and improve the management of this patient group.

More recently, Amato et al. suggested an alternative approach to scale tidal volumes in ARDS patients [13]: the authors hypothesized that the ratio of tidal volume to respiratory system compliance (V_T_/C_RS_) results in an index (driving pressure ΔP), which considers the decreased functional lung capacity during disease. By that, tidal volumes might be more adequately normalized to functional lung size than by the calculation of tidal volumes using body height and PBW alone. Driving pressure ΔP can be calculated at bedside (Plateau pressure–PEEP) and serve as a limit to guide ventilation in order to reduce overstress and overstrain during mechanical ventilation. Sizing tidal volumes with this height independent variable might attenuate the effect of inaccurate definition of height or PBW and serve as a more suitable target goal in mechanical ventilation.

Indeed, Amato and colleagues identified driving pressure as the variable that was most strongly associated with survival in patients with ARDS [13]. Since then, several authors discussed the option of scaling tidal volumes to functional lung size by using driving pressure, but until today, no prospective study has investigated the effect of driving pressure as a primary goal in the ventilation therapy of ARDS patients [14–16].

Therefore, the final effect of the approach using driving pressure remains unclear and further studies are needed to investigate this question. As current guidelines recommend LTV on basis of the PBW there remains the importance of a precise definition of height, which is underlined by evidence suggesting that ALI can be complicated by ventilator-induced lung injury [17,18]. Precise values must be used as errors lead to incorrect results that may increase the risk of death. Unfortunately, most studies do not specify how data of body-height measurement have been collected [2,3,5,19,20]. Consequently, it remains unclear how precise the defined PBW of most studies truly are.

In contrast, the different methods of height determination itself have been well studied: actual body height can be estimated, measured from crown to heel or derived from the length of the forearm, size of lower legs or demispan [9,10,21]. In addition, these studies investigated the accuracy of different height measurement techniques, already identifying visual estimates as being imprecise [5,9]. Bojmehrani et al. compared the accuracy of different measurement techniques in a study collective consisting of 100 patients. Their heights ranged from 159 to 174cm (median 167cm) and by that represent a collective with rather short statures. Like in our study, the authors found large potential errors when PBW was visually estimated. However, none of these prior studies examined whether the use of estimates resulted in a lower rate of LTV, but the authors discussed this as a possible hazard.

As only a minority of patients recognized with ALI receive LTV [2,4,20,22], it is important to investigate why a evidence-based treatment strategy is insufficiently implemented in daily routine. Walkey *et al*. discussed multiple assumed factors explaining this underutilisation, one of which was a shorter stature [11]. Additionally, we identified the technique of visual height estimation to be a possible novel contributor to the underutilisation of LTV, interestingly with a higher risk in shorter patients.

We can provide a complementary explanation for these findings based on systemic bias in height estimation: estimates lean towards the mean of the underlying sample distribution. Petzschner *et al*. proposed a general model that explains systematic errors in magnitude estimation by using Bayesian inference as a mathematical framework that combines prior knowledge with new sensory inputs [23]. The authors suggest this concept might be a general principle in psychophysical judgement that could explain behaviour in any type of magnitude estimation. Therefore, the proposed Bayesian model offers an explanation for our findings: assessors try to optimise their behaviour by allocating estimates within the most likely range of height as driven by experience of a normal height distribution [12]. As a consequence, height-dependent magnitudes such as PBW or tidal volumes are biased towards the expected mean of a normal height distribution. Similar observations can be found in multiple trials from the fields of behavioural science or cognitive science that investigated magnitude estimation and psychophysical judgement. In those studies, estimation of multiple magnitudes was subject to reoccurring characteristic bias: regression effect, range effect, and scalar variability [23–25].

Interestingly, our logistic regression model adjusted for sex, age, profession and years of work experience revealed a 1.74-fold increased risk of overestimating if the assessor was a female (OR 1.74; 95%CI 1.14–2.65; p = 0.01). In contrast, profession or level of training did not affect the accuracy of estimations. As women being generally of a shorter height, further studies are needed to assess whether an assessors´ own stature possibly influences psychophysical judgement and thereby contributes to the systematic error of magnitude estimation.

The present study had several limitations. First, because this was an observational, single-centre study, we could not demonstrate causality between visual estimation of body height and a negative impact on survival. We only simulated ventilator settings and did not observe outcome variables, for example mortality. Nevertheless, to assess survival we refer to the study of Needham *et al*. because it was a robust study providing a three-level categorical model for estimating mortality [2].

Second, possible influences such as missing ventilator variables (e.g. plateau pressure, positive end-expiratory pressure, inspiration time or use of the prone position) were not covered by our analyses.

Third, we undertook multivariate analyses predominantly for male Europeans–the majority of our patients. As a consequence, generalisation with respect to other patient groups is limited.

Finally, we had a small study group recruited from a single tertiary hospital. Our results may not be equivalent to experiences in other medical centres.

## Conclusions

The common clinical practice in most ICUs is to use visual estimates to calculate tidal volumes for ventilation therapy. According to our data, most estimates were significantly inaccurate in comparison to precisely measured results. Our study demonstrates that this practice of body size definition is imprecise and possibly harmful.

Therefore, if applied to calculate tidal volumes, this method potentially increases mortality as it may result in inappropriate mechanical ventilation.

Visual estimation of heights as a reference size for ventilator settings is systematically biased, as estimates are dependent on assessor´s personal experience and subjective judgement, by that implying a high risk of inaccuracy. As a consequence, it should be imperative to accurately measure body height, disregarding any time constraints affecting busy ICUs.

## Supporting information

S1 Dataset Complete dataset of all included patients. (XLSX)
